# Research Progress of Perfluoroalkyl Substances in Edible Oil—A Review

**DOI:** 10.3390/foods12132624

**Published:** 2023-07-06

**Authors:** Yingyi Han, Xueli Cao

**Affiliations:** Beijing Advanced Innovation Center for Food Nutrition and Human Health, Beijing Technology and Business University, Beijing 100048, China; hanyi@btbu.edu.cn

**Keywords:** perfluoroalkyl substances, edible oil, pre-treatment, determination

## Abstract

Perfluoroalkyl substances (PFASs) have been widely used in different types of consumer and industrial applications such as surfactants, household cleaning products, textiles, carpets, cosmetics, firefighting foams, and food packaging because of their good stability and special physicochemical properties of hydrophobicity, oleophobicity, high temperature resistance, etc. Meanwhile, PFASs are considered an emerging organic pollutant due to their persistence and potential toxicity to human health. PFASs occur in edible oil, an important component of the global diet, mainly in three ways: raw material contamination, process contamination, and migration from oil contact materials. Thus, the occurrence of PFAS in edible oils has drawn more and more attention in recent years. In this work, the pertinent literature of the last two decades from the Web of Science database was researched. This review systematically addressed the potential sources, the contamination levels, and the progress of the determination of PFASs in edible oil. It aims to provide a relatively whole profile of PFASs in edible oil, render assistance to minimise human exposure to PFASs, and standardise the detection methods of perfluoroalkyl substances in edible oil.

## 1. Introduction

Since the 1930s, the fluorine chemical industry has grown, resulting in the production and use of perfluoroalkyl substances (PFASs), which are a vast range of man-made aliphatic chemicals. The production of perfluorooctanoic acid (PFOA) by 3M Co. in the 1950s has been the starting point for the development of PFASs, with over 5000 compounds being created in the past 70 years [1,2,3]. PFASs tend to be persistent and toxic under normal environmental conditions because of their strong carbon-fluorine covalent bond [4,5]. A recently published article has suggested that PFAS should be treated as a unified group due to the fact that their perfluorocarbon components are not easily decomposed or take a long time to do so under natural conditions [6].

There have been plenty of published works confirming the toxicological effects of PFASs. PFASs have been confirmed to have genotoxicity [7,8], liver toxicity [9,10], male reproductive toxicity [11,12], as well as neurotoxicity [13,14], developmental toxicity [15,16], immunotoxicity [17,18], endocrine disruption [19,20,21], and other toxicity [22,23]. PFAS exposure has been shown to significantly increase incident mortality for liver cancer, kidney cancer, and testicular cancer [24,25,26]. A higher presence of plasma-PFAS, particularly perfluorobutanoic acid (PFBA), was linked to a more severe COVID-19 prognosis, and this association was still evident even when taking into account variables [27,28]. As an emerging organic pollutant, PFASs have potential risks to human health.

Dietary intake is considered to be the main route of human exposure to PFASs, particularly the consumption of foods with a high protein content because they easily bind to protein [29]. Edible oils (refer to both animal fats and vegetable oils in this paper) of animal origin typically come from high protein sources such as meat and fat, whereas those of plant origin are typically extracted from oil crops. Some oil crops, like soybean, sunflower, and rapeseed, are relatively high in protein (40% for soybean, 21% for sunflower, and rapeseed) [30]. Thus, the raw materials for edible oil can be contaminated with PFAS. PFAS contamination can also be introduced during the processing of edible oils [30]. Furthermore, oil-contact materials such as plastic containers, cookware, or baking paper could lead to PFAS migration to oil [31]. According to the information provided by the U.S. Department of Agriculture, the production volume of edible plant oil, which is an important dietary component, was close to 203 million tonnes in 2019 [32]. Thus, great attention should be paid to the contamination of edible oil with PFAS.

Recently, researchers have been focusing on the potential for PFASs to accumulate in edible oil and the implications this may have on food safety, as well as the behaviour of these compounds in the food supply chain. The findings of the studies demonstrate that these substances are highly persistent in the environment and can accumulate in the body over time, leading to a range of adverse effects [2]. Hence, it is imperative to monitor PFAS levels in edible oils in order to safeguard the health of consumers. To guarantee the safety of edible oils, food manufacturers and regulatory authorities must take steps to reduce the presence of PFASs and other contaminants in their products. This includes proper testing, storage, and handling procedures to ensure that edible oils remain safe for consumption. The determination of PFAS in edible oils is a complex process due to the various factors involved. A suitable pre-treatment of samples and highly sensitive analytical techniques are needed to minimise matrix interference and ensure accurate and reliable results [33,34]. Additionally, the fatty acids abundant in edible oil with a similar carbon skeleton to PFASs can also cause misidentification [35]. These challenges must be taken into consideration in order to ensure accurate and reliable results of the investigation and risk assessment of PFAS contamination in edible oil.

Currently, there are very few reviews on PFAS studies in edible oil. This paper aims to review the categories of PFASs, related legislation, the sources of PFASs in edible oil, and analytical methods for PFASs in edible oil. The current progress and developing trend of PFAS detection techniques in edible oil will also be addressed. The findings of this review would provide insights into strategies to reduce human exposure to these harmful chemicals and provide information for subsequent research and risk assessment.

## 2. Classification

PFASs consist of a chain of varying carbon length, on which all of the hydrogen atoms bound to the carbon chain in the non-fluorinated substances have been replaced by fluorine atoms [36]. Their chemical structure also contains a charged functional group attached at one end [37]. Depending on the different functional groups of substitutes, PFASs can be classified into ionic PFASs and neutral/non-ionic PFASs [38,39], as shown in Table 1. At ambient pH, ionic PFASs, including perfluoroalkyl carboxylic acids (PFCAs), perfluoroalkane sulfonic acids (PFSAs), perfluoroalkane sulfinic acids (PFSIAs), perfluoroalkyl phosphonic acids (PFPAs), perfluoroalkyl phosphinic acids (PFPIAs), and perfluoroalkane sulfonamides (FASAs), are generally negatively charged. Neutral PFASs, usually as precursors of PFASs, remain neutral at pH in water and remain in a non-dissociated state, including perfluoroalkane sulfonyl fluorides (PASFs), perfluoroalkanoyl fluorides (PAFs), perfluoroalkyl iodides (PFAIs), perfluoroalkyl aldehydes (PFALs), perfluoroalkyl esters (PFEs), and fluorotelomer alcohol (FTOHs). The chemical formula of PFASs can be expressed as C_n_F_2n+1_-R, where R is a hydrophilic group that substitutes hydrogen atoms.

It is important to recognise that perfluoroalkane sulfonamido derivatives containing an H atom on the N atom are acidic and can break down into amide anions, the extent of which is determined by the surrounding environment or physiological conditions [36]. Many studies assert that non-ionic PFASs are commonly regarded as precursors of ionic PFASs, and they can be biodegraded and metabolised to PFCAs [39,40].

## 3. Legislation

PFOA, as the first man-made PFAS, was successfully synthesised by 3M Co. of the United States in the 1950s [1]. Subsequently, 3M’s epidemiological research found that those exposed to PFOA had an elevated risk of death from prostate cancer, and another study revealed that perfluorooctane sulfonate (PFOS) exposure was linked to an increased risk of death from bladder cancer [41]. When it was discovered that PFOA and PFOS accumulated in people and the environment, 3M took preventative action and voluntarily started to phase out production of PFOS, PFOA, and PFOS-related products in 2000 [42]. 3M ceased its global production of PFOS, PFOA, and PFOS-related products and replaced them with short-chain PFASs such as perfluorobutanesulfonic acid (PFBS) and PFBA in 2002–2003 [42,43].

To mitigate the health risks of PFASs, governments and industries have strengthened legislation and control measures in recent years by emphasising source control and strengthening product risk management. Some important legislation and policies launched during the last two decades are listed in Table 2. The Stockholm Convention on Persistent Organic Pollutants (POPs) has included PFOS, PFOA, perfluorohexanesulphonic acid (PFHxS), their salts, and associated compounds in its catalogue of controlled substances from 2009 to 2022 [44,45,46]. Significantly, the European Food Safety Authority (EFSA) has changed its PFOA and PFOS tolerance limits multiple times between 2008 and 2020, which indicates that there is growing concern over the health effects of PFASs in Europe [47,48,49]. The maximum levels for the sum of PFOS, PFOA, PFNA, and PFHxS in meat and eggs were set to be 1.3–45 μg kg^−1^ wet weight by the European Union (EU) in 2023 [50]. Similarly, the United States Environmental Protection Agency (USEPA) is also adjusting downward the drinking water health advisories for PFOA and PFOS; hexafluoropropylene oxide dimer acid (GenX) and PFBS (as alternatives to traditional PFASs) were first given the health advisories in 2022 [51,52,53]. The Toxics in Packaging Clearinghouse (TPCH) has proclaimed that packing materials and their components must not incorporate PFASs in 2021 [54]. The Food and Drug Administration (FDA) declared it would phase out certain short-chain PFASs in the food market by 2024 [55]. In Asia, the Ministry of Ecology and Environment of the People’s Republic of China (MEPC) ranks PFOS, its salts, perfluorooctane sulfonyl fluoride (PFOSF), PFOA, its salts, PFOA-related compounds, PFHxS, its salts, and PFHxS-related compounds as key regulated new pollutants in 2023 [56]. The Chemical Substances Control Law (CSCL) of Japan announced that PFOA and its salts were added to the list of Class I Specified Chemical Substances (the import, manufacture, or sale of products containing Class I substances is prohibited) [57].

It can be seen that PFASs are becoming a global concern. However, much of the PFAS legislation is centred on environmental contamination, whereas comparatively fewer regulations address food safety.

## 4. Sources of PFASs in Edible Oil

Given the heightened awareness of PFASs globally, it is especially critical to identify the sources of PFAS pollution in edible oils. Having a thorough knowledge of the causes of PFAS pollution in edible oils can assist in precisely pinpointing the sources of contamination and taking the necessary steps to decrease PFAS contamination and thereby safeguard human health. By recognising the sources of contamination, it is possible to enhance the effectiveness of pollution control, avoid superfluous investments, save resources, and advance sustainable environmental progress.

### 4.1. PFAS Accumulation in Oil Crops

As previously mentioned, PFASs are easily accumulated in protein-rich food matrices. Most of the contamination of oil crops occurred during their growth stage, which will severely affect the safety of edible plant oil [32]. Edible plant oil can be derived from a variety of oil crops such as rapeseed, soybean, peanut, and sesame. Additionally, the protein concentration in soybeans and peanuts was approximately 40% and 28%, respectively, which was even more than that in milk [60]. The PFAS life cycle begins with the primary producer and progresses to the commercial user, consumer, and eventually disposal, all of which involve the release of chemicals into the atmosphere and water and the storage of PFASs in soils for a prolonged period of time [61]. The presence of PFASs in soils has been reported worldwide [62,63,64], while PFOA constituted the main component of PFASs in soil and plants due to its high solubility in water [65]. The accumulation of PFASs could lead to acute toxic effects on growth and development in plant communities [66]. It was reported that soybean and rape can absorb PFOS-K and PFOA from soil by root and transfer them to the stem and leaf; the concentrations of PFOS-K and PFOA in root, stem, and leaf were positively correlated with the concentrations in soil [67], which is consistent with the published result that PFASs could be absorbed and accumulated via plant root from soil and water [65]. Moreover, Tian et al. also confirmed that airborne PFASs and homologs presented in vapour and particulate form could be adsorbed into plants by aerial parts such as foliage and bark [68]. Furthermore, the transport/distribution of PFASs by the plant from the root to above-ground tissues is mostly related to the chain length; the longer-chain PFASs prefer to accumulate in the root, while the shorter-chain compounds prefer to transport to other tissues [65]. As mentioned above, oil crops absorb PFASs from the environment (soil, water, and atmosphere) readily and eventually transfer them to oil products [69].

### 4.2. PFAS Accumulation in Animal Edible Oil Raw Materials

In addition to edible plant oils, edible oils of animal origin are also widely used because of their rich nutrients, such as lard, butter, fish oil, cod liver oil, etc. However, studies conducted both in the field and in the laboratory have revealed that certain PFASs can accumulate in the bodies of predators in wildlife, water, and land environments, as well as in humans [70]. As opposed to lipophilic-bioaccumulative POPs, PFASs are involved in a protein-associated bioaccumulative pathway [71]. PFASs can attach to proteins in the serum of the blood, resulting in high concentrations of these chemicals in both the blood and the blood meals [72]. Therefore, PFASs could enter the food chain through various pathways, mainly through PFAS contamination in animal living environments and feed additives.

Aquatic edible animals from a river-estuary-sea environment that were affected by the fluorochemical industry have been widely reported around the world [73,74,75]. The research conducted in China to investigate the PFCA levels in the edible tissues of 40 aquatic species from the river-estuary-sea environment, affected by a large fluorochemical industrial area, revealed that PFOA was the major contaminant, with concentrations as high as 2161 ng g^−1^ wet weight (found in the freshwater winkle) [75]. Of the 200 North East Arctic cod liver samples tested for 16 PFASs, PFOS was present in the majority of the samples (72%) at concentrations above the limit of quantitation (LOQ) (1.5 μg kg^−1^ wet weight), with the highest level detected being 21.8 μg kg^−1^ wet weight [76]. This is in good accordance with the fact that PFOS and PFCAs are prone to accumulate in the liver [77,78]. However, the PFSAs in cod liver oil are not under supervision, and little is known about them. PFOA and PFOS were also detected in fish samples collected from a local supermarket in Sweden at concentrations of 4.15 pg g^−1^ and 55.3 pg g^−1^, respectively [79].

The consumption of terrestrial animals, such as dairy and meat products, is a major source of edible animal-based oils. The contamination of PFASs in milk (butter-making raw materials) and meat (animal oil-making raw materials) was also frequently reported [80,81]. PFOS and PFOA were detected in milk samples from Greece. Concentrations of PFOS varied from <LOQ to 730 pg g^−1^ and PFOA from <LOQ to 570 pg g^−1^ (LOQ: 500 pg g^−1^) [82]. Analysis of milk samples from Turkey indicated that PFOA was not detected at levels above the reported LOQ of 38 pg g^−1^, yet PFOS was present at concentrations between 544 and 828 pg g^−1^ [83]. Researchers studied the concentrations of seven PFCAs and three PFSAs in milk and milk products from Poland. The most commonly detected was PFOA, followed by PFBA and perfluorohexane sulfonate, on par with perfluorooctane sulfonate. PFBA was the most prominent PFAS present in the studied food items, and it had an average concentration of 13.34 ng g^−1^ [84]. PFOS and PFOA were 100% detected in cow milk, butter, beef, and chicken meat samples; the highest PFOA and PFOS concentrations were found in butter at 9.4 pg g^−1^ and 114 pg g^−1^ [79].

PFASs have also been found in feed and animal-derived food, as well as in the transfer of PFASs through the “feed-to-food” chain [85,86]. Hence, PFAS contamination in animal feed should be taken into consideration. Ninety-two commercial fishmeal samples from the most important fishmeal-producing countries were collected for evaluating PFAS levels, and the results showed that Σ16 PFCAs ranged from 6.29 to 84.5, 1.42–52.0, 2.47–45.3, 1.06–42.1, and 1.02–38.8 ng g^−1^ in the U.S., China, Europe, South America, and Southeast Asia, respectively. Noteworthy, the presence of short-chain PFCAs (e.g., PFBA and PFBS) in fishmeal was found for the first time in the study [85]. Researchers from the same group also collected the most commonly used animal protein supplement feeds (blood meal, meat meal, feather meal, soybean meal, and dried grains with solubles), with concentrations of Σ16 PFCAs ranging from undetectable to 37.1 ng g^−1^ dw (dry weight) (average: 7.23 ng g^−1^ dw) [72]. The investigation of the occurrence of PFASs in cow feed samples from nine Chinese provinces revealed concentrations in the range of 0.99–144 ng g^−1^ dw (7.68 ng g^−1^ dw) and the PFBA dominating 34.0% of PFASs in feed [87]. In eighteen different lab animal feeding materials, PFOS, PFHxS, PFOA, and short-chain PFCAs (C < 6) had the highest detection levels and frequencies across all samples. PFAS levels found in feed were as high as 215.6 μg kg^−1^ dw [88]. It is of great concern that feed exposure to PFASs has not drawn enough attention. Meanwhile, investigations conducted recently have revealed an increase in the amount of short-chain PFCAs and PFSAs found in the environment, which may be due to the restriction of long-chain PFASs, causing a reduction in the use of long-chain PFASs and a heightened focus on short-chain PFASs.

### 4.3. Contamination of PFASs during Edible Oil Production

**Figure 1:**
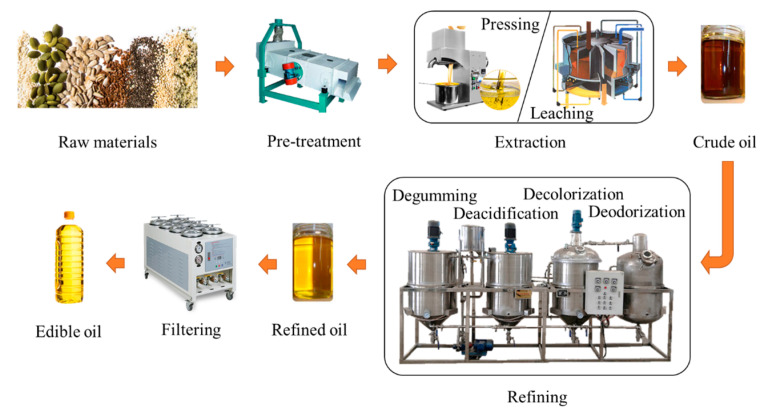
Edible oil production process.

Most common edible oils are plant-derived through intricate manufacturing steps, which increases the risk of PFAS contamination. The primary emphasis here is on the PFAS pollution that occurs during plant-derived oil production. The production of edible oil mainly includes three parts: extraction, refining, and filtering [89], as shown in Figure 1. High temperatures can cause the breakdown of PFAS-containing materials, releasing more of these chemicals into the solvent, which in turn can dissolve in numerous organic solvents and ultimately into the product. Therefore, PFASs are easily introduced during extraction and refining processes involving high temperatures and organic solvents. The unrefined oil produced by mechanical pressing or solvent extraction of oil crops is called crude oil, which is not edible. During the pressing process, physical processes such as high-temperature baking and crushing may increase the concentration of environmental pollutants in oil products caused by the grinding machine being contaminated with PFAS-made detergent and lubricant, as well as PFASs from the plastic interface of the grinding machine. The solvent extraction is to extract the oil raw material by soaking it in organic solvent oil, which could also introduce PFASs [90]. It has been suggested that the higher concentration of C8-chain-length PFASs detected in the crude oil may be caused by the degradation of the precursor material into stable C8-chain-length PFASs and by the contamination of the crude oil by the processing apparatus [91].

The refining process of edible oil mainly includes degumming, deacidification, decolorization and deodorization (Figure 1) [92,93]. PFASs are used in the production of various chemicals that are commonly used in the edible oil industry, such as surfactants and emulsifiers. Surfactants and emulsifiers are usually added to the oil during the refining process to improve its texture, taste, and appearance [94,95,96]. Furthermore, both deacidification and decolorization are performed at higher temperatures, which may also increase the possibility that PFASs dissolve in edible oil products.

The lack of research on the introduction of PFAS in the manufacturing of edible oils makes additional investigations necessary.

### 4.4. Migration from Oil Contact Materials to Edible Oil

A broad range of PFASs are used in paper and plastic, and they are commonly used to package high-fat content and convenience foods nowadays due to their non-hydrophilic and non-lipophilic properties [97]. Direct contact between the food contact materials and food could facilitate the migration of these PFASs into food products [98,99,100]. The migration of PFASs from contact materials to edible oil products occurs in the following two ways. First, oil-contact packaging, storage, and transport processing [90,101,102], particularly the use of plastic containers, can allow contaminants to leach into oil products depending on the contact time with the packaging materials [103,104]. Second, the product oil is usually ingested after being heated in contact with fluorocarbon resin-coated frying pans, baking utensils, and non-stick baking papers [31]. Research revealed that the concentration of PFOA in empty cooking pans increased up to 75 µg kg^−1^ after heat treatment [90]. The presence of PFOA and PFOS was determined in polytetrafluoroethylene (PTFE)-coated non-stick cookware sold in Turkey [105]. It was noted that FTOHs could migrate from paper bowls to oil, with migration efficiencies ranging from 0.04 to 2.28%; however, the efficiency of migration decreased as the carbon chain length of the FTOHs increased [106]. Therefore, routine monitoring and risk assessment of PFAS in oil are necessary.

**Figure 2:**
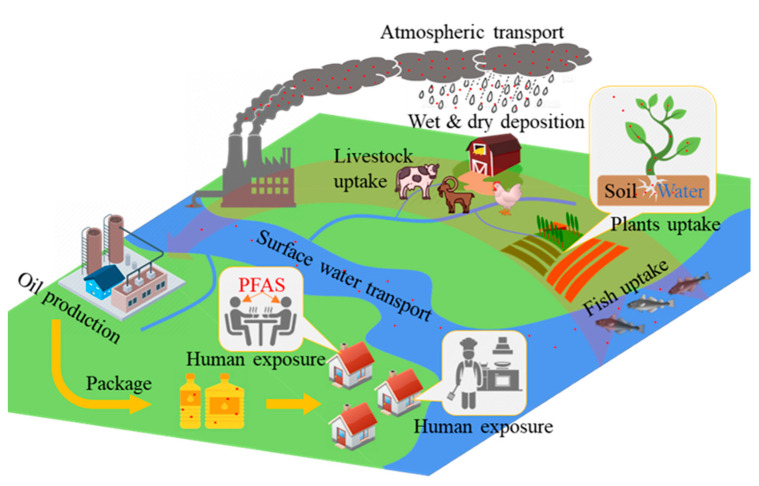
The life cycle of PFASs in edible oil from the primary producer to human exposure.

It is essential to take the PFAS life cycle into account. These compounds are long-lasting in the environment, entering it through production and manufacturing processes, consumer use, and disposal. Once introduced to the environment, PFASs can remain in the atmosphere, aquifers, and the ground and can become concentrated in the tissues of living organisms, which could potentially be used as sources of edible oil. Edible oils are unavoidably contaminated with PFAS as a result of the use of fluorinated consumer products at the manufacturing, processing, and packaging stages, thus raising the probability of human exposure. The flow of PFASs from the primary producer to the product oil and the human body is shown in Figure 2.

## 5. PFAS Contamination in Edible Oil

Plenty of the published literature reports PFAS contamination in food as the major exposure pathway for the general population [107], especially for potable water, vegetables, fruits, milk, seafood, and meat [61,81,108]. Research into the effects of PFASs in edible oils has been ongoing for several years due to the possibility that edible oils could introduce PFAS contamination via various pathways and as an important route of human exposure to PFASs. Table 3 shows the concentrations of selected PFASs in various edible oils from several countries. The selected PFASs were mainly ionic PFASs; only isolated studies showed detectable levels of non-ionic PFASs.

A total of sixteen different PFASs were determined in margarine sold in the Norwegian market, and six PFASs, namely PFHxS, PFOS, PFBA, perfluorovaleric acid (PFPeA), perfluorohexanoic acid (PFHxA), and PFOA, were identified at concentrations above the LOQ. The highest concentrations of 51, 51, and 12 pg g^−1^ were observed in PFBA, PFPeA, and PFOA, respectively. The concentrations of other selected PFASs were below the given limit of detection (LOD). The LODs for that investigation were comparatively low, ranging from 1.6 pg g^−1^ (PFBS) to 13 pg g^−1^ (perfluoroundecanoic acid (PFUnDA)/perfluorododecanoic acid (PFDoDA)). The study showed PFOA to be more concentrated than PFOS (2.3 pg g^−1^), which was contrary to what other research had suggested [109].

A total of 14 PFASs were measured in vegetable oil and butter, which were sold in Dutch retail store chains with nationwide coverage. For butter, PFHxA, perfluoroheptanoic acid (PFHpA), PFOA, PFNA, perfluorodecanoic acid (PFDA), PFDoDA, PFHxS, and PFOS were detected between LODs and LOQs; PFOS showed the highest concentrations of 33 pg g^−1^. In vegetable oil, only PFHpA was detected above LOD at 1 pg g^−1^ [110].

The presence of 18 PFASs in the olive oil purchased in Catalonia, Spain, was assessed. PFOS was the only PFAS detected at a concentration of 1.1 pg g^−1^ amongst the 18 PFASs, while the others were detected at levels below the LODs, ranging from 0.22 (PFDS) to 140 pg g^−1^ (PFHpA and PFOA) [112].

Swedish researchers established disparities in the levels of PFASs (PFCAs and PFASs) in fatty food items obtained from a Swedish food market between 1999, 2005, and 2010. The findings indicated similar levels of concentration with minimal variation between years. Results from the year 2010 indicated that PFOS, PFHxA, and PFUnDA were present at concentrations of 13, 4.3, and 5.8 pg g^−1^, respectively. The concentration of other compounds was below the LOD, with a range from 2.3 pg g^−1^ (PFHpA) to 3.9 pg g^−1^ (PFHxA) [114]. The butter samples obtained from Sweden also applied for PFHxS, PFOA, and PFOS detection; the concentrations ranged from 2.6 to 18 pg g^−1^, 6.8–56 pg g^−1^, and 8.1–21.3 pg g^−1^, respectively [79].

PFOA and PFOS were measured in olive and peanut oils sold in the supermarket in Siena (central Italy). The result revealed that the concentration of PFOA and PFOS was below the LOD of 500 pg g^−1^ [115]. Similarly, no analysed PFASs were identified in the edible oils and butter from the Czech Republic that exceeded the LOQ values (1400 pg g^−1^ for PFHxA and 2700 pg g^−1^ for PFHpA) [116].

The presence of 10 selected PFASs (seven PFCAs and three PFSAs) was measured in commonly consumed fats and oil samples (sunflower oil, rapeseed oil, margarine, and a mix of margarine and butter) collected from the Polish market, and all ten PFASs tested were detected. PFOA was found with the highest (100%) detection frequency in the analysed PFCAs. For the PFSA family, PFOS was the most commonly detected. The concentration of PFOA ranged from 53 pg g^−1^ in olive oil to 1962 pg g^−1^ in sunflower oil, while the concentration of PFOS was below 51 pg g^−1^, as specified for the olive oil sample. PFBS was found only in rapeseed oil samples, with concentrations ranging from 3.0 to 5.0 pg g^−1^ [30].

To date, the research project on edible oil in China encompasses four areas, including Guangzhou, Guizhou, Shandong, and Beijing. PFOA was detected but could not be quantified, and PFOS was not detected in the oil samples (peanut oil and mixed vegetable oil) sold in Guangzhou, China [117]. A total of 18 PFASs were analysed in different types of commercial edible oils purchased from local supermarkets in Guiyang, China. PFOS, PFNA, PFHxS, and PFOA were the predominant pollutants among the 18 PFASs at concentrations of 1.93 ng g^−1^, 6.76 ng g^−1^, 0.36 ng g^−1^, and 0.15 ng g^−1^, respectively [69]. A total of seven PFCAs (C6–C12) were detected in all three cod liver oils obtained from the Shandong local market, with concentrations that ranged from 2.1 to 40 μg L^−1^ [118]. The contamination of five PFCAs in different edible vegetable oils sold in Beijing, China, was reported. The main target chemicals in these oils were PFOA and PFDA, which were discovered in six oil samples with concentrations between 6 pg g^−1^ and 2458 pg g^−1^. Long-chain compounds such as PFOA can get up to 2018 pg g^−1^. The short-chain PFCAs, including PFBA and PFHxA, were also detected at concentrations of 37 and 588 pg g^−1^ in blended oil, respectively [35]. The concentrations of five target PFCAs in sesame oil were relatively high among the analysed samples, which is consistent with Yang’s study in 2015 [60]. Similarly, 18 PFASs (PFCAs, PFSAs, and PFASs) were analysed in six edible plant oils with different manufacturing processes and brands purchased from supermarkets in Beijing. The PFOA concentration was the most prominent out of the 18 PFASs tested, ranging from below the LOD to 500 pg g^−1^ with a detection rate of 92%. The highest concentration detected was 4640 pg g^−1^ of PFNA in blend oil [60]. Recently, the contamination of PFAIs in edible oil samples obtained from supermarkets in Beijing, China, was investigated. Perfluorobutyl iodide (PFBI) and perfluorohexyl iodide (PFHxI) were detected in seven of the twelve oil samples, and their concentrations ranged from 629.8 to 3053 pg g^−1^. Perfluorooctyl iodide (PFOI) was found in five of twelve oil samples in the concentration range of 212.9 to 2626 pg g^−1^. Perfluorodecyl iodide (PFDeI) and perfluorododecyl iodide (PFDoI) were detected only in camellia seed oil at 226.8 and 560.6 pg g^−1^, respectively [119].

It can be said that the contamination of PFAS in edible oils is worldwide, and C6–C10 PFCAs and PFSAs were extensively present in the edible oils. In contrast, a majority of the research conducted on PFOA in PFCA and PFOS in PFSA revealed greater contamination of PFOA than PFOS, whereas only a small number of studies investigated short-chain PFASs (PFBA and PFBS) and non-ionic PFASs (PFAIs). Information remains limited, but some existing studies have already suggested that these highly fluorinated alternatives (short-chain PFASs, etc.) may not be less persistent, less bioaccumulative, or less toxic as intended [34,120,121,122]. Given the growing prevalence of short-chain and novel PFAS products, they deserve more in-depth study.

## 6. Pre-Treatment Methods for PFAS Analysis in Edible Oil

It is essential to evaluate the levels of PFAS in edible oils due to possible contamination. Monitoring edible oils for PFASs assists in identifying the presence of the compounds, enabling a more accurate evaluation of the oils’ safety, and assists regulatory authorities in identifying any quality issues so they can take prompt action to effectively manage food safety hazards.

**Figure 3:**
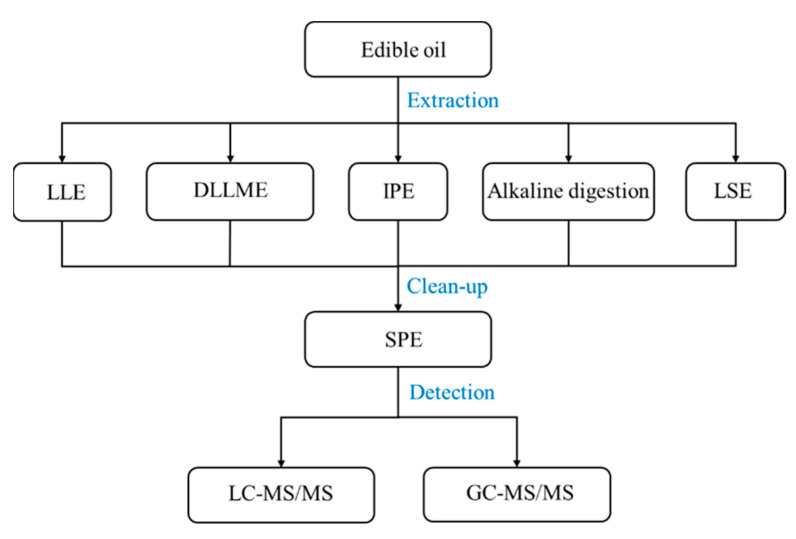
Schematic diagram of the analysis method for PFASs in edible oil.

The analysis of PFASs in edible oils is incredibly difficult due to the trace levels of analytes and the high fat content in the matrix, especially fatty acids, which are ubiquitous in edible oils, have a similar carbon skeleton to PFASs, and can lead to significant matrix effects and misidentification [118,123]. Hence, pre-treatment strategies are important for the extraction and clean-up of target compounds. At present, the extraction of PFASs from oil samples is mainly carried out by liquid-liquid extraction (LLE), dispersive liquid-liquid microextraction (DLLME), ion-pairing extraction (IPE), alkaline digestion, and liquid-solid extraction (LSE). These methods have their own advantages and disadvantages for the extraction of PFASs in different samples, so it is crucial to choose suitable extraction methods for different matrices. In addition, solid phase extraction (SPE) is usually employed for further enrichment and clean-up of targets after sample extraction to enhance detection efficiency and minimise the matrix effect during the detection (Figure 3).

### 6.1. Liquid-Liquid Extraction (LLE)

LLE, also known as solvent extraction, is a classical pre-treatment method that takes advantage of differences in the partition coefficients between targets and impurities in two incompatible solvents [124]. Good extraction efficiency and recovery for PFASs in oil make LLE a distinct advantage, but it has a large toxic and harmful solvent consumption, which does not meet green chemistry criteria [125].

A one-step reversed-phase liquid-liquid extraction was processed, using a mixture of basified water/methanol (1:1, *v*/*v*, containing 0.5% NH_3_H_2_O) as the aqueous system and dichloromethane as the non-polar system, for separating PFOA and PFOS from cooking oil and extracting them into the aqueous system. The instrumental LOQs of PFOA and PFOS were 0.01 ng mL^−1^ [117].

### 6.2. Ion Pair Extraction (IPE)

IPE is a technique for the selective extraction of polar (i.e., acidic/basic) compounds from aqueous samples into an organic phase with the assistance of counter-ions, comprising different hydrophobicities as ion-pairing reagents [126].

The IPE was employed using methyl tert-butyl ether (MTBE) and subsequent solid phase extraction clean-up on Florisil and graphitised carbon for 11 target PFASs determination. The recovery of fat is 62–91%. PFOS, PFUnDA, and PFHxA were detected in fat, and the concentrations were 13, 5.8, and 4.3 pg g^−1^, respectively [114]. PFOS and PFOA were extracted using an IPE procedure and measured using high-performance liquid chromatography (HPLC) with electrospray ionisation (ESI) tandem mass spectrometry. However, PFOS and PFOA were not detected in olive and peanut oils [115].

### 6.3. Dispersive Liquid-Liquid Microextraction (DLLME)

DLLME, which is the miniaturised version of liquid-liquid extraction in that the amount of organic solvent used is dramatically reduced and shows a very high enrichment factor compared to other liquid- or even solid-phase extraction methods, is an easy and quick method for the extraction and purification of organic compounds present at trace levels in aqueous samples [127]. The primary benefit of DLLME is that it has a greater contact area than LLE, which enhances, speeds up, and optimises the extraction [128].

The traditional DLLME technique involved the rapid addition of a mixture of a water-insoluble extraction solvent dissolved in a water-soluble solvent to an aqueous sample. Magnetic deep eutectic solvents (DESs) are recent approaches to DLLME, either with or without the need for dispersion solvents and without the need for centrifugation to break the dispersion [129]. The superparamagnetic nanofluid, based on a new choline chloride/1-(o-tolyl) biguanide DES system, was dispersed into edible oil for direct extraction of five PFCAs from edible oils. The pre-treatment process was performed in 15 min, recoveries ranged from 90 to 109%, and the LOD was 0.3–1.6 pg g^−1^. In six out of the ten edible oil samples. PFOA and PFDA were the predominant target compounds and detected at concentrations between 6 pg g^−1^ and 2458 pg g^−1^. Long-chain compounds like PFOA can get up to 2018 pg g^−1^, and short-chain PFCAs such as PFBA and PFHxA were also detected with concentrations ranging from 37 to 588 pg g^−1^ [35].

### 6.4. Alkaline Digestion

Alkaline digestion of lipids and proteins before extraction was suggested to achieve an accurate and reliable measurement of PFASs. Alkaline digestion could reduce the matrix effect and improve extraction efficiency. However, this method generally requires shaking for hours, prolonging the pre-treatment time, which is not conducive to the rapid detection of pollutants [130]. For alkaline digestion, the same solvents were used; the sample matrix was digested using sodium hydroxide or potassium hydroxide solutions [34].

PFASs were extracted from freeze-dried margarine using alkaline digestion (sodium hydroxide in methanol), followed by SPE using weak anion exchange (WAX) and additional clean-up with Styrene-divinylbenzene (ENVI)-carb. PFHxS, PFOS, PFHxA, and PFOA were detected in margarine, and the concentrations were 1.3, 2.3, 2.5, and 12 pg g^−1^, respectively [109]. The same alkaline digestion extraction methods were also performed in freeze-dried olive oil, and only PFOS was detected at a level of 1.1 pg g^−1^ [112].

### 6.5. Liquid-Solid Extraction (LSE)

LSE is the separation of components in a solid mixture using a solvent by partition of analytes between the two involved phases, the matrix and the extractant, which is essentially a mass transfer process in which the solute is transferred from the solid phase to the liquid phase only [128]. LSE is usually used for extracting solid or semi-solid samples such as butter and margarine. An LSE extraction technique was used for PFAS extraction from margarine based on extraction with a mixture of tetrahydrofuran and water (*v*/*v*:3:1). The combination of a WAX resin and Envi-carb SPE in-line was found to be effective in reducing matrix effects in the clean-up process. A total of eight PFASs were detected in butter as follows: PFOS 33 pg g^−1^, PFHxA 20 pg g^−1^, PFOA 16 pg g^−1^, PFHxS 16 pg g^−1^, PFDA 6 pg g^−1^, PFHpA 5 pg g^−1^, PFNA 2 pg g^−1^, and PFDoDA 2 pg g^−1^ [110].

### 6.6. Solid Phase Extraction (SPE)

SPE is used for purification and enrichment of targets, and it is usually employed after extraction in the processing of complex oil samples. SPE is performed by the interaction (ion exchange, physical adsorption, hydrophobic action, etc.) of a solid adsorbent between the target compound and the adsorbent for the purpose of separating the target compound from the sample matrix and the interfering compound [131]. However, this pre-treatment method requires a large cost in purchasing expensive solid-phase extraction cartridges.

By using acetonitrile extraction, extract modification by water, and keeping the sample refrigerated at 5 °C before subjecting it to SPE, the sample preparation process was significantly simplified to decrease analysis costs. The validity of the approach was confirmed and applied to actual edible oil samples. The recovery of butter and oil is 37–118% and 45–120%, respectively. The LOQ of PFAS was 1.4–2.7 ng g^−1^ [116]. The PFASs in sunflower oil were extracted by tetrahydrofuran-water solvent, followed by WAX resin and Envi-carb SPE. The recovery ranged from 88 to 110%, and the LOD ranged from 2.5 to 60 pg g^−1^ [111].

#### 6.6.1. Dispersive Solid-Phase Extraction (d-SPE)

D-SPE is a novel sample pre-treatment technique developed from traditional SPE techniques and is commonly used to extract target compounds from samples. The technique is based on the addition of a sorbent to the sample, allowing the desired components to attach to it, and then the target compounds are eluted using a solvent to achieve enrichment and separation of the target compounds. D-SPE is popular in analytical chemistry due to its minimal solvent consumption and its uncomplicated, speedy, and effective operation [132].

PFASs were extracted by acetonitrile and purified by gel permeation chromatography (GPC) and d-SPE using graphitised carbon black (GCB) and octadecyl (C18). The recoveries of PFASs ranged from 60% to 129%, and the detected PFAS concentrations in the oils ranged from below the LOD to 4.64 μg kg^−1^. It should be noted that the highest level of PFNA detected was 4.64 μg kg^−1^ in blend oil, and this is likely because PFNA is not a chemical that is strictly regulated worldwide [60].

#### 6.6.2. Magnetic Solid-Phase Extraction (MSPE)

The fact that the magnetic or magnetisable sorbent can be disseminated in the sample solution to improve the interfacial area between the sorbent and sample gives MSPE numerous advantages over traditional SPE techniques. The magnetic sorbent is included in the sample solution in MSPE technology. An external magnetic field is used to separate the magnetic sorbent from the sample solution after the target component has been absorbed into it. Following that, a suitable solvent is used to elute the extracted analytes from the sorbent. Finally, the magnet separates the eluent from the sorbent and introduces it to analytical tools [133,134].

A sensitive method was used to analyse the PFAIs, combining an UiO-66-NH_2_@DES-based MSPE with a gas chromatography-mass spectrometer (GC-MS). Under optimised conditions, the proposed approach demonstrated great sensitivity with methodology recovery (74.9–111%) and a LOD of 2.81–34.3 pg g^−1^. The technique worked well for assessing PFAIs in various food oils, and the targets were found in a number of samples with concentrations between 212.9 and 3053 pg g^−1^ [119].

### 6.7. QuEChERS (Quick, Easy, Cheap, Effective, Rugged, and Safe)

The QuEChERS approach is a SPE technique based on LLE and d-SPE, which are frequently used for the extraction of organic compounds in food items [34]. The method relies on the combination of solvents and salts to separate the analyte from the sample into acetonitrile [135].

PFAS analysis of oil products was performed using the QuEChERS method. Samples were accurately weighed, targets were extracted using acetonitrile by sonication and vortex following NaCl and MgSO_4_ treatment, and the supernatant was cleaned up with ENV SPE bulk sorbent. PFOA was the most commonly detected compound in fat and oil food samples, with a detection frequency of 100%, and PFBA was found at the highest levels. The LOQ varied from 0.002 to 0.075 ng g^−1^ depending on the perfluoroalkyl compound [30].

## 7. Determination of PFAS in Edible Oil

PFASs could not be detected by UV or fluorescence detectors because there are no chromophores in PFASs. Hence, GC-MS/MS and liquid chromatography (LC)-MS/MS have become the dominant determination methods for target PFASs.

In general, the ionic PFASs were usually analysed using LC-MS/MS, and the non-ionic PFASs were analysed using GC-MS/MS [136]. To this day, the instrumental analysis of PFASs in edible oil is focused on ionised PFAS detection, especially for PFSAs and PFCAs, as shown in Table 3. Therefore, LC-MS/MS is the main instrumental analysis method. With the advent of modern analytical instruments, more sensitive analytical methods have been developed, such as high-performance liquid chromatography (HPLC) and ultra-performance liquid chromatography (UPLC) combined with mass spectrometry (MS), such as HPLC-MS/MS, UPLC-MS/MS, and UPLC-QTOF-MS, etc. As shown in Table 4, UPLC-QTOF-MS displayed the highest accuracy with a LOD of 0.3–1.6 pg g^−1^, followed by UPLC-MS/MS (LOD: 2.3–5.4 pg g^−1^). Although these instruments are expensive, they have outstanding advantages in the analysis of trace analytes with more analytical modes, higher sensitivity, higher selectivity, and less background interference.

Non-ionic PFASs, the precursors of PFASs, are widely used in various fields as emerging POPs. Therefore, instrumental analysis of non-ionic PFASs should also be given great attention. Most non-ionic PFASs are reported to be volatile and could be analysed by GC-MS/MS [136]. Four FTOHs were determined in the childcare environment using GC-MS, and the LOD is 20–70 pg g^−1^ [39]. PFAIs were also analysed by GC-MS/MS [119,136]. To date, there are few studies on the GC-MS/MS analysis of non-ionic PFASs in edible oils.

Although chromatography offers the sensitivity and precision necessary for analysis, the intricate procedures, expensive apparatus, and personnel expertise required make it difficult to exploit its full testing potential, with a cost per sample of around USD 200–300 [137]. Recently, emerging low-cost detection methods have been developed for PFAS detection, such as optical assays like fluorescent detection (LOD 27.8 nM for PFOS) [138], colorimetric detection (LOD 8.6 nM for PFOS) [139], and electrochemical-based sensor assays (LOD 70 ng L^−1^ for PFOS and PFOA) [140]. Photoelectrochemistry (PEC) was also used as a detection technique with the potential to increase sensitivity and miniaturisation [141,142]. These methods could serve as a strategy for the invention of novel detection techniques for PFAS in edible oils.

## 8. Conclusions

The potential health and environmental risks of PFASs have caused an increasing focus on their monitoring by various analytical techniques. Up to this point, PFAS detection has been limited to traditional ionic PFAS analysis in a simple food matrix. Traditional PFASs (i.e., long-chain PFCAs, PFSAs, etc.) are being strictly restricted in production and use all over the world. Subsequently, emerging PFASs (non-ionic PFASs and short-chain PFASs) are introduced into markets. Edible oils, as an important route of exposure to PFASs, deserve special attention. The pool of new PFAS compounds is rapidly growing and now consists of numerous substances with diverse chemical compositions, volatility, and solubility, the physical, chemical, and toxicological properties of which are still unknown and could result in potentially hazardous exposure.

For the safety of edible oils, faster, simpler, and more effective analytical methods for PFASs are imperative. For the detection of PFASs in edible oils, LLE or LSE extraction is usually performed, followed by SPE for further enrichment, and the target PFASs are analysed using GC/LC-MS/MS. It is essential to develop a universal pre-treatment process for all the target PFASs that have a broad spectrum of polarity. Multiple interaction strategies may be introduced to maximise their extraction/absorption effectiveness.

Despite the progress made in research into the effects of PFASs on edible oil, there is still much to be investigated. For instance, safe human exposure levels need to be assessed, and effective control measures need to be explored in food products. Additionally, in-depth research on the environmental effects of these compounds, their interaction with other components of the food system, their migration and transformation into the food system, and their impact on food safety is still essential.

## Figures and Tables

**Table 1:** Classification of PFASs.

PFASs	Classification	R	Examples	Structural Formula	CAS
Ionic PFASs	Perfluoroalkyl carboxylic acid, PFCAs	–COOH	Pentadecafluorooctanoic acid, PFOA	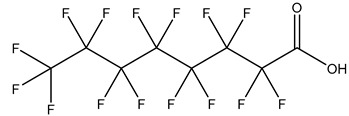	335-67-1
	Perfluoroalkane sulfonic acids, PFSAs	–SO_3_H	Perfluorooctane sulfonic acid, PFOS	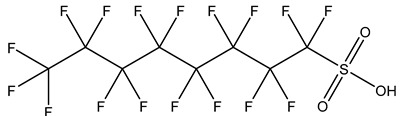	1763-23-1
	Perfluoroalkane sulfinic acids, PFSIAs	–SO_2_H	Perfluorooctane sulfinic acid	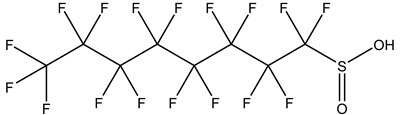	647-29-0
	Perfluoroalkyl phosphonic acids, PFPAs	–P(=O)(OH)_2_	Perfluorooctyl phosphonic acid, PFOPA	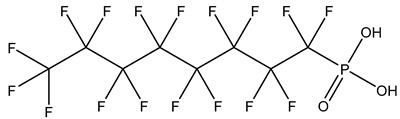	40143-78-0
	Perfluoroalkyl phosphinic acids, PFPIAs	–P(=OH)(C_n_F_2n+1_)	Bis(heptadecafluorooctyl) phosphinic acid	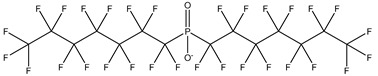	500776-69-2
	Perfluoroalkane sulfonamides, FASAs	–SO_2_NH_2_	Perfluorooctane sulfonamide, FOSA	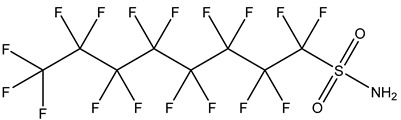	754-91-6
Non-Ionic PFASs	Perfluoroalkane sulfonyl fluorides, PASFs	–SO_2_F	Perfluorooctane sulfonyl fluoride, PFOSF	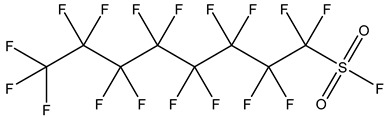	307-35-7
	Perfluoroalkanoyl fluorides, PAFs	–COF	Perfluorooctanoyl fluoride	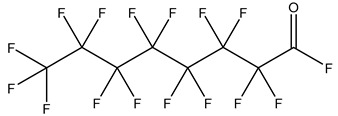	335-66-0
	Perfluoroalkyl iodides, PFAIs	–I	Perfluorooctyl iodide, PFOI	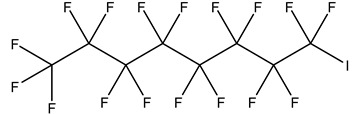	507-63-1
	Perfluoroalkyl aldehydes, PFALs	–CHO	(Perfluorooctane)-1-carbaldehydle	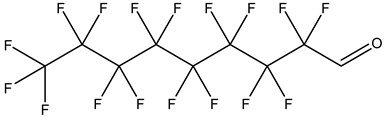	63967-40-8
	Perfluoroalkyl esters, PFEs	–COOR	Perfluorooctanoic acid methyl ester	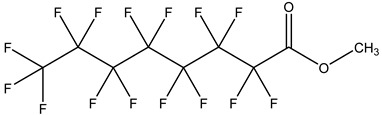	376-27-2
	Fluorotelomer alcohol, FTOHs	–OH	1H,1H,2H,2H-Perfluorooctanol, 6:2 FTOH	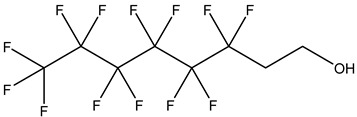	647-42-7

**Table 2:** Important legislation and policies on PFAS launched during the last two decades.

District	Year	Regulation	Restriction	Reference
Global	2009	Stockholm Convention on POPs	PFOS and its salts were listed in Annex B (restriction).	[44]
	2019	Stockholm Convention on POPs	PFOA, its salts, and PFOA-related compounds were listed in Annex A (elimination).	[45]
	2022	Stockholm Convention on POPs	PFHxS, its salts, and PFHxS-related compounds were listed in Annex A (elimination).	[46]
Europe	2008	EFSA	The tolerable daily intake (TDI) of 150 ng kg^−1^ bw.d^−1^ (body weight/day) for PFOS and 1500 ng kg^−1^ bw.d^−1^ for PFOA was established.	[47]
	2018	EFSA	The tolerable weekly intake (TWI) for PFOS is 13 ng kg^−1^ bw. wk^−1^ (body weight/per week) and for PFOA is 6 ng kg^−1^ bw. wk ^−1^.	[48]
	2020	EFSA	The TWI of 4.4 ng kg^−1^ b.w. for the sum of PFOA, perfluorononanoic acid (PFNA), PFHxS, and PFOS was suggested.	[49]
	2023	EU	The maximum levels for the sum of PFOS, PFOA, PFNA, and PFHxS in meat and eggs were set to be 1.3–45 μg kg^−1^ wet weight.	[50]
USA	2009	USEPA	The minimum risk levels of PFOA and PFOS in drinking water were set to be 0.4 and 0.2 μg L^−1^.	[51]
	2015	USEPA	The minimum risk levels of PFOA and PFOS in drinking water were set to be 0.07 μg L^−1^. When both PFOA and PFOS are found in drinking water, the combined concentrations of PFOA and PFOS should be below 0.07 μg L^−1^.	[52]
	2021	TPCH	TPCH has proclaimed that packing materials and their components must not incorporate PFASs.	[54]
	2021	FDA	The FDA declared it would phase out certain short-chain PFASs in the food market by 2024.	[55]
	2022	USEPA	The minimum risk levels of PFOA, PFOS, GenX chemicals, and PFBS in drinking water were advised to be 0.000004, 0.00002, 0.01, and 2 μg L^−1^.	[53]
	2023	USEPA	The USEPA announced the proposed action of PFOS, PFOA, PFHxS, PFNA, GenX chemicals, and PFBS into the National Primary Drinking Water Regulation (NPDWR).	[58]
Asia	2019	MEPC	Prohibit the production, circulation, use, and import or export of PFOS, its salts, and PFOSF except for acceptable uses.	[59]
	2023	MEPC	PFOS, its salts, PFOSF, PFOA, its salts, PFOA-related compounds, PFHxS, its salts, and PFHxS-related compounds were included in the list of key regulated new pollutants (version 2023).	[56]
	2021	CSCL	PFOA and its salts were added to the list of Class I Specified Chemical Substances (the import, manufacture, or sale of products containing Class I substances is prohibited).	[57]

**Table 3:** Content of PFSAs and PFCAs in edible oils (pg g^−1^).

District	Matrix	PFSAs				PFCAs													Reference
		C4^a^	C6^a^	C7^a^	C8^a^	C4^b^	C5	C6^b^	C7^b^	C8^b^	C9	C10	C11	C12	C13	C14	C16	C18	
Norway	Margarine	<1.6	**1.3**	-	**2.3**	**51**	**51**	**2.5**	<5.6	**12**	<13	<8.6	<16	<16	-	-	-	-	[109]
Netherlands	Vegetable oil	<0.9	<2	-	<3	<32	<28	<3	**1**	<3	<0.1	<0.6	<2	<1	-	-	-	-	[110]
	Butter	<3	**16**	-	*33*	<31	<43	**20**	5	*16*	**2**	**6**	<3	**2**	-	-	-	-	
Spain	Sunflower oil	-	ND	-	ND	ND	-	ND	-	ND	ND	ND	ND	-	-	-	-	-	[111]
	Olive oil	<1.2	<0.7	-	*1.1*	<43	<5.9	<14	<140	<140	<38	<3.8	<14	<4.2	<6.1	<6.2	<48	<41	[112]
Finland	Fish oil	-	-	-	ND	-	-	-	-	ND	-	-	ND	-	ND	ND	-	-	[113]
Sweden	Fats (butter, margarine, cooking oil, and mayonnaise)	-	<2.3	-	**13**	-	-	*4.3*	<2.3	<5.4	<3.0	<3.6	*5.8*	<2.3	<2.3	<2.3	-	-	[114]
	Butter	-	*2.6–18*	-	**8.1–21.3**	-	-	-	-	**8.1–56**	-	-	-	-	-	-	-	-	[79]
Italy	Olive oil	-	-	-	<500	-	-	-	-	<500	-	-	-	-	-	-	-	-	[115]
	Peanut oil	-	-	-	<500	-	-	-	-	<500	-	-	-	-	-	-	-	-	
Czech Republic	Edible oils	*ND-1600*	*ND-1800*	-	*ND-1700*	-	-	*ND-1400*	*ND-2700*	*ND-1500*	*ND-2400*	*ND-1700*	*ND-1700*	*ND-2100*	-	-	-	-	[116]
	Butter	*ND-1600*	*ND-1800*	-	*ND-1700*	-	-	*ND-1400*	*ND-2700*	*ND-1500*	*ND-2400*	*ND-1700*	*ND-1700*	*ND-2100*	-	-	-	-	
China Guangzhou	Cooking oil	-	-	-	*ND-20* pg mL^−1^	-	-	-	-	*ND-20* pg mL^−1^	-	-	-	-	-	-	-	-	[117]
China Guiyang	Rapeseed oil	-	140	ND	390	130	120	-	-	90	940	-	-	90	-	-	100	160	[69]
	Blended oil	-	240	ND	450	110	ND	-	-	80	1770	-	-	ND	-	-	ND	ND	
	Peanut oil	-	170	ND	290	ND	ND	-	-	ND	ND	-	-	ND	-	-	ND	ND	
	Corn oil	-	120	ND	210	ND	ND	-	-	ND	ND	-	-	ND	-	-	110	140	
	Sunflower oil	-	ND	ND	220	ND	ND	-	-	ND	ND	-	-	ND	-	-	ND	ND	
	Lard oil	-	230	210	330	ND	ND	-	-	-	710	-	-	90	-	-	100	ND	
	Beef tallown	-	130	ND	290	ND	ND	-	-	-	ND	-	-	ND	-	-	110	ND	
China Beijing	Blended oil	-	-	ND	-	-	-	*ND-60*	ND	*ND-430*	*ND-4640*	ND	-	-	-	-	-	-	[60]
	Soybean oil	-	-	ND-20	-	-	-	*ND-440*	*ND-40*	*130–160*	*20–470*	*ND-40*	-	-	-	-	-	-	
	Peanut oil	-	-	ND	-	-	-	*80–100*	ND	*180–240*	ND	ND	-	-	-	-	-	-	
	Sesame oil	-	-	*20–30*	-	-	-	*ND-490*	*50–80*	**150–500**	*30–1060*	*160–510*	-	-	-	-	-	-	
	Corn oil	-	-	ND	-	-	-	*ND-450*	ND	**150–170**	*ND-20*	*ND-40*	-	-	-	-	-	-	
	Sunflower oil	-	-	ND	-	-	-	**400–500**	ND	**400–500**	ND	**400–600**	-	-	-	-	-	-	
	Olive oil	-	-		-	<1.6	-	ND	-	ND	-	ND	-	ND					[35]
	Sesame oil	-	-		-	**405**	-	**112**	-	**2018**	-	**2458**	-	**1841**					
	Corn oil	-	-		-	ND	-	ND	-	<0.5	-	17	-	7					
	Camellia seed oil	-	-		-	<1.6	-	57	-	21	-	9	-	5					
	Soybean oil	-	-		-	ND	-	ND	-	ND	-	8	-	7					
	Blended oil (80% Corn; 20% sesame)	-	-		-	**432**	-	ND	-	**8**	-	ND	-	ND					
	Blended oil (70% Corn; 30% sesame)	-	-		-	**588**	-	**37**	-	**21**	-	**6**	-	<0.3					
	Vegetable oil	-	-		-	ND	-	ND	-	<0.5	-	ND	-	ND					
China Shandong	Cod liver oil	-	-	-	-	-	-	ND	**2100–6200**	**40,000**	**9000**	**8200**	**3400–5800**	**3400–6300**					[118]
	Fish Oil	-	-	-	-	-	-	ND	ND	ND	ND	ND	ND	ND	-	-	-	-	
Poland	Sunflower oil	ND	ND	-	ND	1062	ND	ND	ND	**640**	565	ND	-	-	-	-	-	-	[30]
	Rapeseed oil	**3**	ND	-	**16**	ND	ND	ND	250	**110**	ND	ND	-	-	-	-	-	-	
	Olive oil	ND	ND	-	ND	**962**	ND	**49**	ND	**27**	ND	ND	-	-	-	-	-	-	
	Margarine	ND	ND	-	ND	ND	ND	ND	ND	**250**	ND	ND	-	-	-	-	-	-	
	Mix of margarine and butter	ND	ND	-	ND	ND	ND	ND	ND	**270**	ND	ND	-	-	-	-	-	-	

C4^a^: PFBS; C6^a^: PFHxS; C7^a^: PFHpS; C8^a^: PFOS; C4^b^: PFBA; C5: PFPeA; C6^b^: PFHxA; C7^b^: PFHpA; C8^b^: PFOA; C9: PFNA; C10: PFDA; C11: PFUnDA; C12: PFDoDA; C13: PFTrDA; C14: PFTeDA; C16: PFHxDA; and C18: PFOcDA; **Bold**: concentrations above LOQ; *Italic*: concentrations above LOD but below LOQ; <: concentration below the given LOD; ND: not detectable; and -: not tested.

**Table 4:** Different analytical methods for PFASs in edible oils.

Extraction	Extraction Solvent/Material	Clean-Up	Clean-Up Material	Recovery (%)	Analytical Instrumentation	LOD (pg g^–1^)	Reference
LLE	Basified water/methanol, dichloromethane	-	-	-	LC-MS/MS	10–2500 pg mL^– 1^	[117]
	Acetonitrile, n-pentane	SPE	DSC-18 SPE cartridge (Sigma-Aldrich)	37–120	HPLC-MS/MS	1400–2700	[116]
	Acetonitrile	GPC+DSPE	C18, GCB	60–129	LC-ES-MS/MS	4–400	[60]
	Acetonitrile	d-SPE	ENV SPE bulk sorbent	72–104	micro-HPLC-MS/MS	2–75	[30]
	Basified methanol/water (1:1, *v*/*v*, containing 1% NH_3_H_2_O)	MSPE	Fe_3_O_4_@SiO_2_@ Quaternary ammonium (QTA)	85.0–98.5	LC-MS/MS	1.5–20	[118]
	Methanol	MSPE	Fe_3_O_4_@UiO-66-NH_2_@DES	74.9–111	GC-MS/MS	2.81–34.3	[119]
DLLME	Superparamagnetic nanofluid		DES based nano Fe_3_O_4_ fluid	90–109	UPLC-QTOF-MS	0.3–1.6	[35]
Ion pair extraction	MTBE	SPE	Florisil and graphitised carbon	62–91	UPLC-ESI-MS/MS	2.3–5.4	[114]
	MTBE	-	-	-	HPLC-ESI-MS/MS	-	[115]
Alkaline digestion	Sodium hydroxide in methanol	SPE	WAX, ENVI-carb	-	UPLC-ESI- MS/MS	-	[109,112]
LSE	Tetrahydrofuran and water	SPE	A weak anion exchange resin and ENVI-carb	-	LC-EI-MS/MS	-	[110]

## Data Availability

Not applicable.
